# Novel method for an optimised calculation of modal analysis of girder bridge decks

**DOI:** 10.1038/s41598-022-16606-4

**Published:** 2022-07-21

**Authors:** Alvaro Gaute-Alonso, David Garcia-Sanchez, Óscar Ramón Ramos-Gutierrez

**Affiliations:** 1 Grupo de Instrumentación y Análisis Dinámico de Estructuras (GiaDe), University of Cantabria, Santander, Spain; 2 TECNALIA Basque Research and Technology Alliance (BRTA), Derio, Spain; 3 Department of Structural and Mechanical Engineering, University of Cantabria, Santander, Spain

**Keywords:** Civil engineering, Information technology

## Abstract

A correct modal analysis of girder bridge decks requires a correct characterisation of the deformation of their cross-section, governed by the longitudinal bending of the girders and the transverse bending of the slab. This paper presents a novel method that allows the modal analysis of girder bridge decks by applying a matrix formulation that reduces the structural problem to one degree of freedom for each girder: the deflection at the centre of the beam span. A parametric study is presented that analyses the structural response of 64 girder bridge decks. The study compares the dynamic structural response obtained by the proposed method with that obtained by traditional grillage calculation methods. The method is experimentally contrasted by a dynamic load test of a full-scale girder bridge. As a result of the analysis, the proposed method reflects adequate convergence with the experimental dynamic structural response. The use of the proposed novel analysis method contributes to the intelligent modelling process for the analysis of the dynamic behaviour of bridges opening the way to easily feed a Digital Twin accelerating the demands of the Decision Support System in real time.

## Introduction

Modal analysis of structures is a technique that consists of obtaining their frequencies and modes of vibration. This technique makes it possible to obtain information on the structure beyond that provided by static analysis methodologies and has become an optimal methodology in the experimental analysis of structures, allowing the early detection of damage or pathologies based on the variation of their frequencies and modes of vibration^1–4^. Experimental modal analysis makes it possible to obtain the dynamic characteristics of structures in places that are difficult to access without the need for auxiliary support elements such as inspection trucks, working platforms at different heights or shear or demolition elements^5,6^.

Girder bridges are a structural typology commonly used in the design of road and railway bridges. The modal analysis of this type of structure has traditionally been solved by using finite element structural models, such as grillage models^7–12^. The need for a calculation method to determine the dynamic characteristics of girder bridge decks, such as their frequencies and modes of vibration, which allows continuous real-time feedback of the models used in pathology detection campaigns from the experimental analysis of their dynamic response, is one of the motivations of the authors for the development of the research work that has given rise to this article.

In engineering there is always a need to reduce the degrees of freedom in computational models for structural evaluation for different reasons^13^: reduction of computational demand, conceptualisation of the physical problem and interpretation of results to standardise methodologies. Some authors use interface constraint modes and deformed substructural modes for model reduction when high accuracy is needed. In some cases, it may be interesting to perform additional reductions after reducing the substructural degrees of freedom, and this is usually done by reducing the interface degrees of freedom. However, this is only necessary when accuracy is preferable to computational time efficiency or interoperability for digital twinning. Particularly, the originality of the method presented in this paper lies in the use of a virtual model, which reflects the transverse stiffness of the girder bridge deck and the torsional stiffness of the longitudinal girders composing the girder bridge deck for easy implementation in a digital predictive modelling system or an early warning system, i.e.

## Traditional methods for girder bridge deck analysis

**Figure 1:**
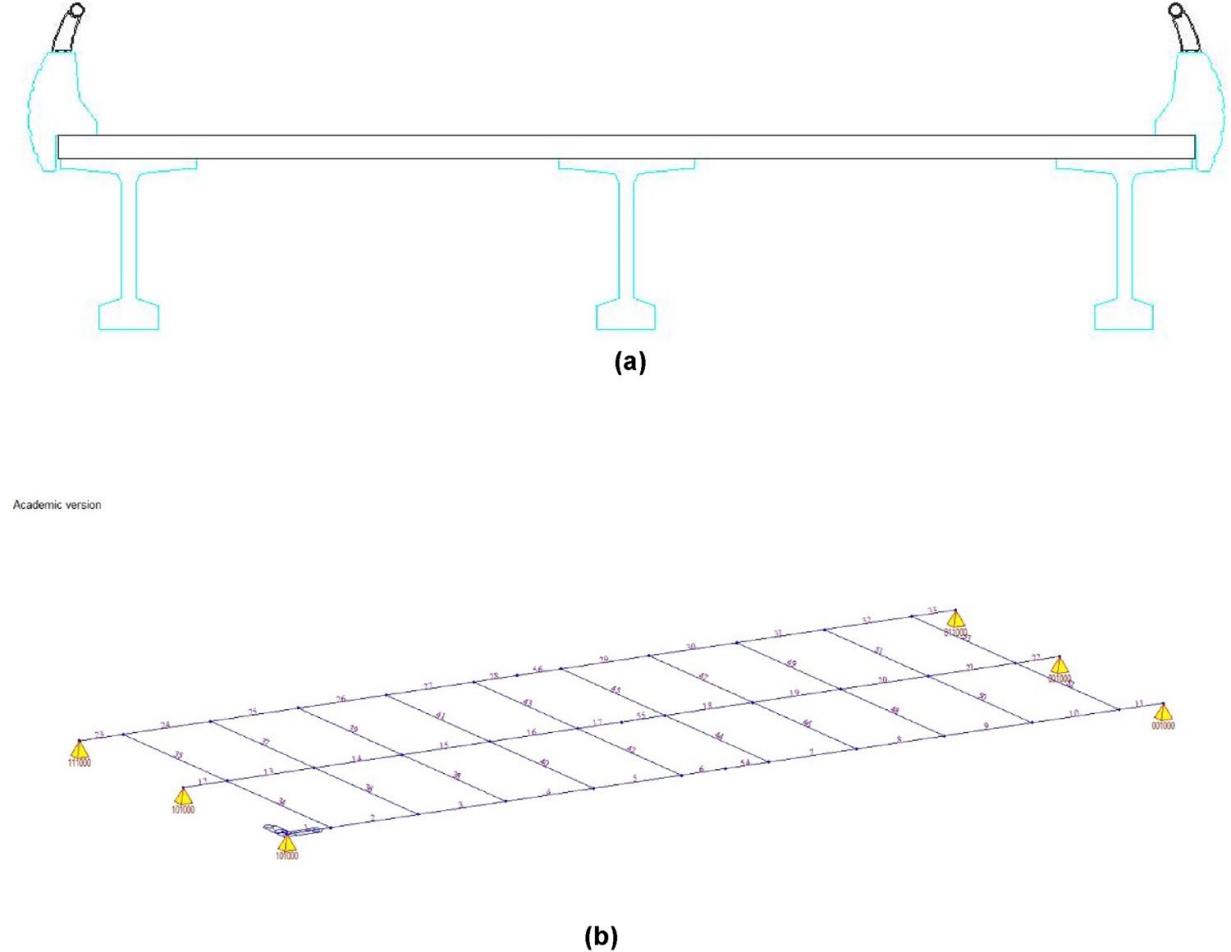
Grillage discretization: (**a**) girder bridge deck cross section; (**b**) structural grillage model.

Structural grillage models began to be used for cross-section deformation analysis of beam bridge decks in the 1960s^14^. These models divide the girder bridge deck into longitudinal and transverse girders (Fig. 1). The longitudinal girders are responsible for providing the longitudinal bending stiffness of the deck, considering as many longitudinal girders as there are girders in the analysed girder bridge deck. The structural section of each of the longitudinal girders will be the resultant of the section composed of the analysed girder and the effective width of the top slab collaborating with that girder^15,16^. The transverse distribution of the loads between the different longitudinal girders of the structural model is given by the transverse girders and the torsional stiffness of the longitudinal girders. The structural cross-section of the transverse girders corresponds to a rectangular cross-section with a depth equivalent to the slab thickness and a width according to the discretisation used in the grillage model.

The use of spatial grillage structural models makes it possible to obtain the structural response of girder bridges, as well as their frequencies and modes of vibration, adequately showing the transverse distortion of the girder bridge and the distribution of the longitudinal bending of the deck between each of the longitudinal girders that compose it. However, this type of model involves a complex and time-consuming analysis and requires the use of specific structural calculation programs, which makes it interesting to use simplified methods to optimise the start of the design process.

## Proposed method for modal analysis of girder bridges

**Figure 2:**
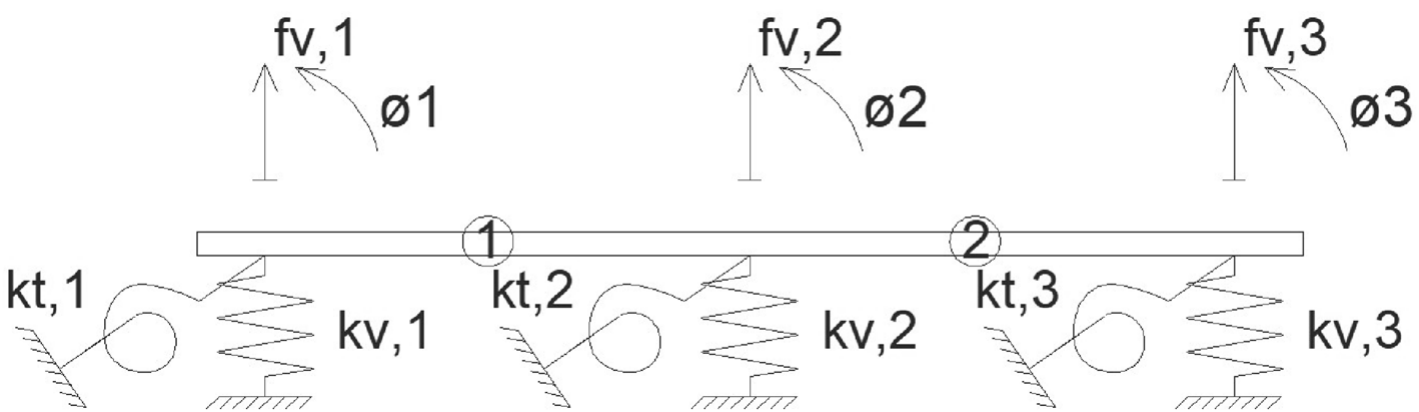
Proposed method model for cross-sectional distribution on a girder bridge deck.

The authors propose the use of a simplified matrix method based on the use of a virtual model, which reflects the transverse stiffness of the girder bridge deck by supporting the top slab on a series of springs that provide the bending stiffness Eq. (1) and torsional stiffness Eq. (2) of the longitudinal girders composing the girder bridge deck (Fig. 2).

1 $$K_{v,i} = \frac{{48 \cdot EI_{i} }}{{L^{3} }}$$

2 $$K_{t,i} = \frac{{2 \cdot GJ_{i} }}{L}$$

where EI_i_ = longitudinal bending stiffness of girder "i"; GJ_i_ = longitudinal torsional stiffness of girder "i"; L = bridge support span.

The proposed method initially considers 2 degrees of freedom for each longitudinal girder of the deck: (1) the deflection and (2) the rotation of the deck slab at the centre of the girder span. Figure 2 represents the schematic of the structural model for a girder bridge deck composed of three longitudinal girders. The matrix approach that solves the structural problem of the transverse distribution of the live loads between the different girders composing the deck is given by the matrix Eqs. (3), (4) and (5).

3 $$\overline{{K_{e} }} = \frac{{EI_{e} }}{{L_{e}^{3} }}\left( {\begin{array}{*{20}c} {\begin{array}{*{20}c} {12} & {6L_{e} } \\ {6L} & {4L_{e}^{2} } \\ \end{array} } & {\begin{array}{*{20}c} { - 12} & {6L_{e} } \\ { - 6L_{e} } & {2L_{e}^{2} } \\ \end{array} } \\ {\begin{array}{*{20}c} { - 12} & { - 6L_{e} } \\ {6L} & {2L_{e}^{2} } \\ \end{array} } & {\begin{array}{*{20}c} {12} & { - 6L_{e} } \\ { - 6L_{e} } & {4L_{e}^{2} } \\ \end{array} } \\ \end{array} } \right) = \left( {\begin{array}{*{20}c} {\overline{{K_{11,e} }} } & {\overline{{K_{12,e} }} } \\ {\overline{{K_{21,e} }} } & {\overline{{K_{22,e} }} } \\ \end{array} } \right)$$

4 $$\overline{{K_{Bi} }} = \left( {\begin{array}{*{20}c} {k_{v,i} } & 0 \\ 0 & {k_{t,i} } \\ \end{array} } \right)$$

5 $$\left( {\begin{array}{*{20}l} {\begin{array}{*{20}c} {P_{1} } \\ {M_{t,1} } \\ \end{array} } \hfill \\ {\begin{array}{*{20}c} {P_{2} } \\ {M_{t,2} } \\ \end{array} } \hfill \\ {\begin{array}{*{20}c} {P_{3} } \\ {M_{t,3} } \\ \end{array} } \hfill \\ \end{array} } \right) = \left( {\begin{array}{*{20}l} {\overline{{K_{11,1} }} + \overline{{K_{B1} }} } \hfill & {\overline{{K_{12,1} }} } \hfill & {\begin{array}{*{20}c} 0 & 0 \\ 0 & 0 \\ \end{array} } \hfill \\ {\overline{{K_{21,1} }} } \hfill & {\overline{{K_{22,1} }} + \overline{{K_{11,2} }} + \overline{{K_{B2} }} } \hfill & {\overline{{K_{12,2} }} } \hfill \\ {\begin{array}{*{20}c} 0 & 0 \\ 0 & 0 \\ \end{array} } \hfill & {\overline{{K_{21,2} }} } \hfill & {\overline{{K_{22,2} }} + \overline{{K_{B3} }} } \hfill \\ \end{array} } \right) = \left( {\begin{array}{*{20}c} {\begin{array}{*{20}c} {f_{v1} } \\ {\theta_{1} } \\ \end{array} } \\ {\begin{array}{*{20}c} {f_{v2} } \\ {\theta_{2} } \\ \end{array} } \\ {\begin{array}{*{20}c} {f_{v3} } \\ {\theta_{3} } \\ \end{array} } \\ \end{array} } \right)$$

where EI_e_ = transverse bending stiffness of the top deck slab element "e"; L_e_ = length of the top deck slab element "e".

**Figure 3:**
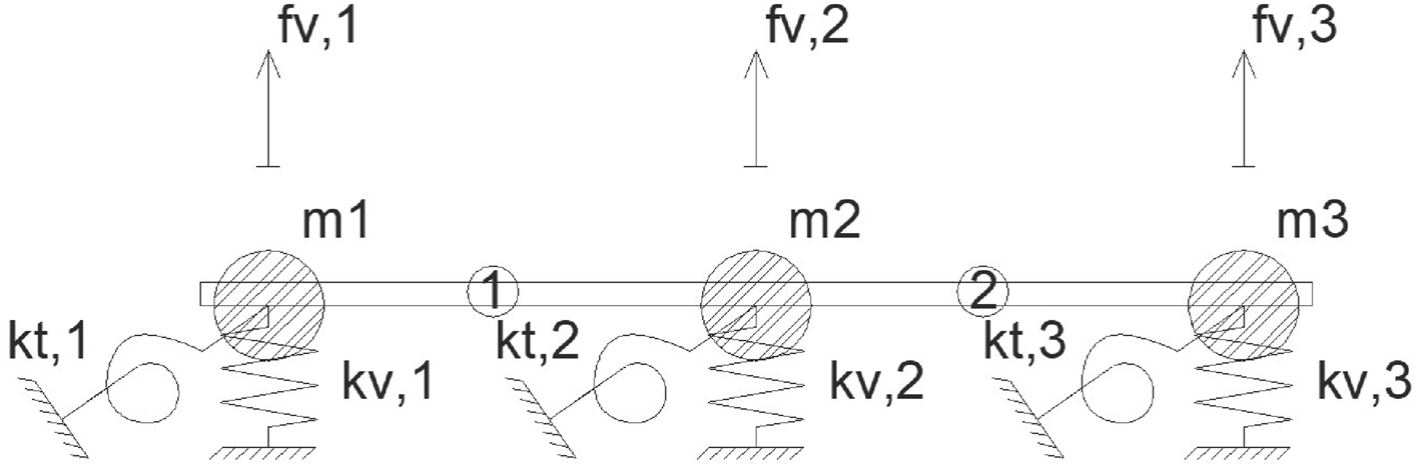
Structural model of concentrated masses.

In this proposal for simplified modal analysis of girder bridge decks, a concentrated mass dynamic structural model is used in which only one degree of freedom is considered for each longitudinal girder forming the girder bridge (Fig. 3). The degrees of freedom considered correspond to the vertical displacement experienced by the centre of the span of each of the longitudinal girders, for which it is necessary to condense the degrees of freedom associated with the torsional rotation of the centre of the span of the longitudinal girders and the bending rotation of the transverse slab. This operation of condensing the degrees of freedom associated with the rotations into the degrees of freedom associated with the vertical movements is known as static condensation^17–20^. To apply the static condensation artifice Eq. (7), it is necessary to rearrange the stiffness matrix of the simplified structural model, so that the degrees of freedom associated with the vertical movement appear first and finally the degrees of freedom associated with the rotation Eq. (6).

6 $$\left( {\begin{array}{*{20}c} {\begin{array}{*{20}c} {P_{1} } \\ {P_{2} } \\ \end{array} } \\ {\begin{array}{*{20}c} {P_{3} } \\ {M_{t,1} } \\ \end{array} } \\ {\begin{array}{*{20}c} {M_{t,2} } \\ {M_{t,3} } \\ \end{array} } \\ \end{array} } \right) = \left( {\begin{array}{*{20}c} {\overline{{K_{VV} }} } & {\overline{{K_{V\theta } }} } \\ {\overline{{K_{\theta V} }} } & {\overline{{K_{\theta \theta } }} } \\ \end{array} } \right) = \left( {\begin{array}{*{20}c} {\begin{array}{*{20}c} {f_{v1} } \\ {f_{v2} } \\ \end{array} } \\ {\begin{array}{*{20}c} {f_{v3} } \\ {\theta_{1} } \\ \end{array} } \\ {\begin{array}{*{20}c} {\theta_{2} } \\ {\theta_{3} } \\ \end{array} } \\ \end{array} } \right)$$

7 $$\overline{{K_{DD} }} = \left( {\overline{{K_{VV} }} - \overline{{K_{V\theta } }} \cdot\overline{{K_{\theta \theta } }}^{ - 1} \cdot\overline{{K_{\theta V} }} } \right)$$

where $\overline{{K }_{DD}}$ = condensed stiffness matrix in the degrees of freedom associated with the vertical movement of the section centre of the longitudinal girders.

The concentrated masses associated with the vertical movement experienced by the centre of section of the longitudinal girders of the deck "m_i_" are the result of the sum of the following values: (a) half of the mass associated with each longitudinal girder; (b) half of the mass associated with the part of the top slab gravitating on each longitudinal girder Eq. (8). By means of the dynamic equilibrium in free vibrations Eq. (9) at the initial instant of motion, the matrix equation Eq. (10) is obtained, which allows obtaining the frequencies and eigenmodes functions of vibration of the girder bridge deck^21–24^. The columns of the eigenmode function matrix "Ø_n_" correspond to the deformation of the girder deck cross-section associated with each of the analysed natural modes of vibration. The eigenmode function associated with each longitudinal girder "Ø_n,Gi_" is approximated by a sinusoidal function Eq. (11).

8 $$m_{i} = \int\limits_{0}^{L} {m \cdot \left[ {\sin \left( {\frac{\pi \cdot x}{L}} \right)} \right]^{2} \cdot dx = m \cdot \frac{L}{2}}$$

9 $$\overline{M}\cdot\vec{\ddot{u}} + \overline{C}\cdot\vec{\dot{u}} + \overline{{K_{DD} }} \cdot\vec{u} = \vec{0}$$

10 $$\left( {w_{n}^{2} \cdot\overline{I} + \overline{M}^{ - 1} \cdot\overline{{K_{DD} }} } \right)\cdot\overline{\emptyset } = \vec{0}$$

11 $$\emptyset_{n,Gi} = {{\sin \left( {\frac{\pi \cdot x}{{{\raise0.7ex\hbox{$L$} \!\mathord{\left/ {\vphantom {L N}}\right.\kern-\nulldelimiterspace} \!\lower0.7ex\hbox{$N$}}}}} \right) \cdot \emptyset_{n} \left( i \right)} \mathord{\left/ {\vphantom {{\sin \left( {\frac{\pi \cdot x}{{{\raise0.7ex\hbox{$L$} \!\mathord{\left/ {\vphantom {L N}}\right.\kern-\nulldelimiterspace} \!\lower0.7ex\hbox{$N$}}}}} \right) \cdot \emptyset_{n} \left( i \right)} {N = ceil\left( \frac{n}{NB} \right)}}} \right. \kern-\nulldelimiterspace} {N = ceil\left( \frac{n}{NB} \right)}}$$

where m_i_ = point mass associated with degree of freedom "i"; m = mass per linear metre gravitating on longitudinal girder "i"; $\overline{M}$ = Matrix of concentrated masses; $\overline{C }$ = Structural damping matrix; $\overrightarrow{u}$ = Motion vector; w_n_ = Angular pulsation associated with mode "n"; $\overline{\varnothing }$ = Eigenmode function matrix; Ø_n_ = Eigenmode function associated with mode "n"; Ø_n,Gi_ = Eigenmode function associated with longitudinal girder "i" and mode "n"; NB = Number of longitudinal girders.

**Figure 4:**
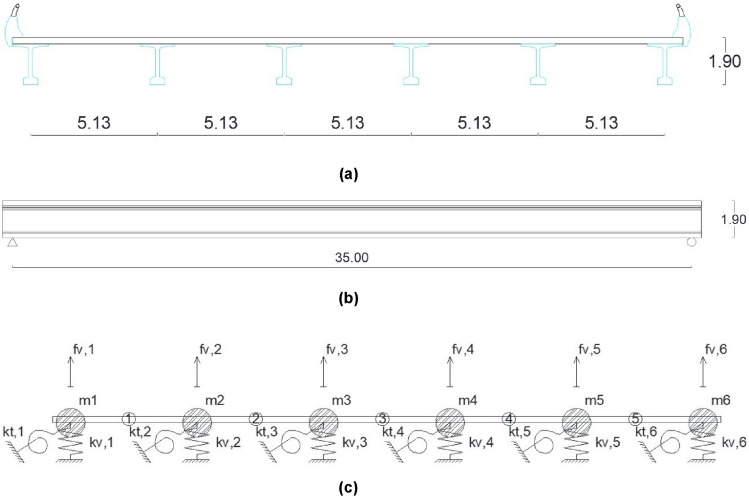
Structural model of a girder bridge with six longitudinal girders: (**a**) cross-section; (**b**) longitudinal profile; (**c**) proposed structural model.

**Figure 5:**
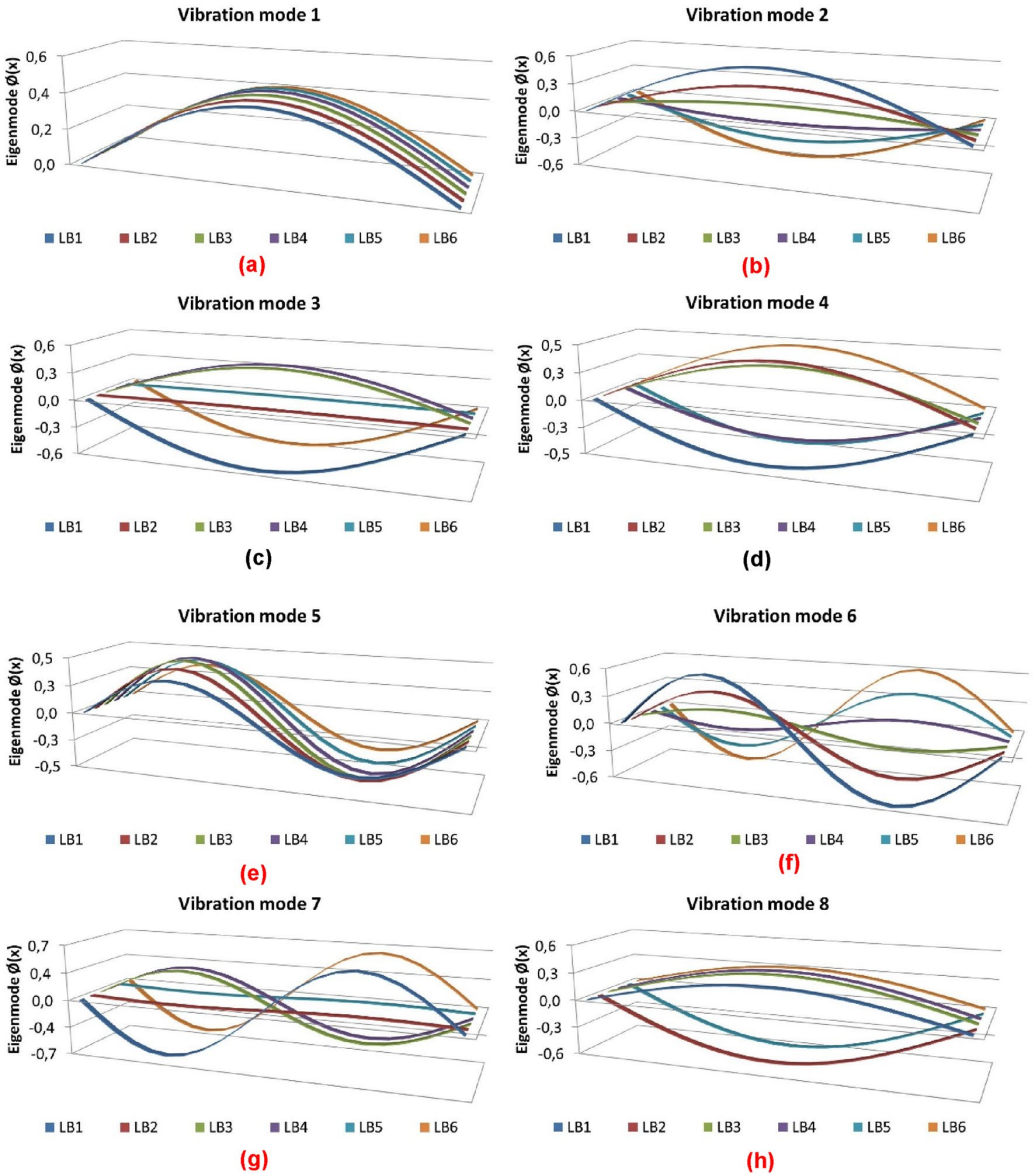
Modal analysis of a girder bridge with 6 longitudinal girders: (**a**) vibration mode 1; (**b**) vibration mode 2; (**c**) vibration mode 3; (**d**) vibration mode 4; (**e**) vibration mode 5; (**f**) vibration mode 6; (**g**) vibration mode 7; (**h**) vibration mode 8.

The proposed simplified method analyses the modes of vibration whose number "n" is greater than the number of longitudinal beams of the deck (n ≥ NB) by means of the matrix equations Eqs. (8) and (9) posed for a girder bridge with equivalent support spacing "Leq = L/N". The numbering of the modes of vibration is defined by their structural stiffness and the mobilised mass. The fundamental modes of vibration of the girder bridge deck correspond to the most flexible and mass mobilising modes, associated with a lower vibration frequency. Figures 4 and 5 show the structural model and shape functions of the eigenmodes of a girder bridge with six longitudinal girders with a support span of 35 m, a longitudinal girder spacing of 5.13 m and a depth of 1.9 m, respectively.

## Parametric study of the dynamic structural response in girder bridge decks

The authors present a parametric study that analyses the dynamic structural response of a total of 64 girder bridge decks, so that all possible combinations of the variables considered in the parametric study are analysed (Table 1). The parametric study compares the dynamic structural response obtained by the proposed method with the structural response obtained by traditional spatial grillage calculation methods. In the spatial grillage model, beam elements with six degrees of freedom have been used and each longitudinal girder has been discretised into 10 sub-elements.

**Table 1:** Variables analysed in the parametric study.

Lenght (m)	Depth (m)	Number of girders
20	1.3	3
25	1.5	4
30	1.7	5
35	1.9	6

**Figure 6:**
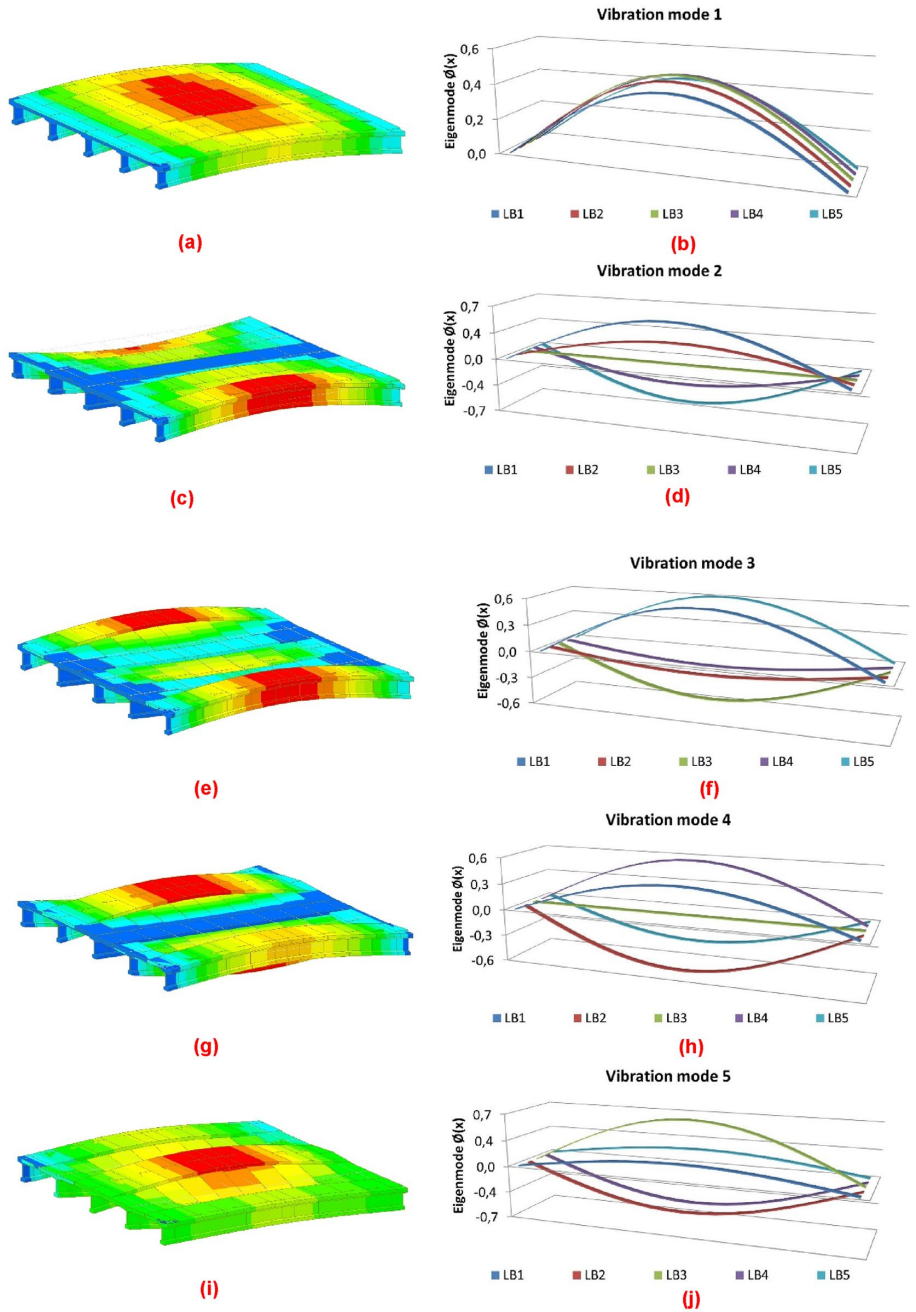
Results of the modal analysis of a five-girder deck: (**a**) mode 1 traditional methods; (**b**) mode 1 proposed method; (**c**) mode 2 traditional methods; (**d**) mode 2 proposed method; (**e**) mode 3 traditional methods; (**f**) mode 3 proposed method; (**g**) mode 4 traditional methods; (**h**) mode 4 proposed method; (**i**) mode 5 traditional methods; (**j**) mode 5 proposed method.

The parametric analysis reflects an adequate convergence of the structural response of the proposed method with respect to that obtained using the traditional grillage calculation methods. The divergence between the structural response obtained using the two types of calculation models analysed is calculated by dividing the difference between the value of the natural frequencies obtained by one and the other method by the value of the natural frequencies obtained by the traditional structural grillage methods. After analysis of 64 calculation models, the average divergence of the structural models is less than 10%, with the average divergence in the fundamental mode of vibration being less than 3%. Figure 6 and Table 2 show a comparison between the results of the modal analysis of a five-girder bridge deck obtained by the traditional grillage calculation methods and the method proposed by the authors.

**Table 2:** Results of the modal analysis of a five-girder deck.

	Traditional methods	Proposed method	Divergence (%)
f1 (Hz)	6.60	6.75	2
f2 (Hz)	6.87	6.75	2
f3 (Hz)	7.41	7.07	5
f4 (Hz)	8.50	8.79	3
f5 (Hz)	10.50	12.52	16

### Discussion of results

**Figure 7:**
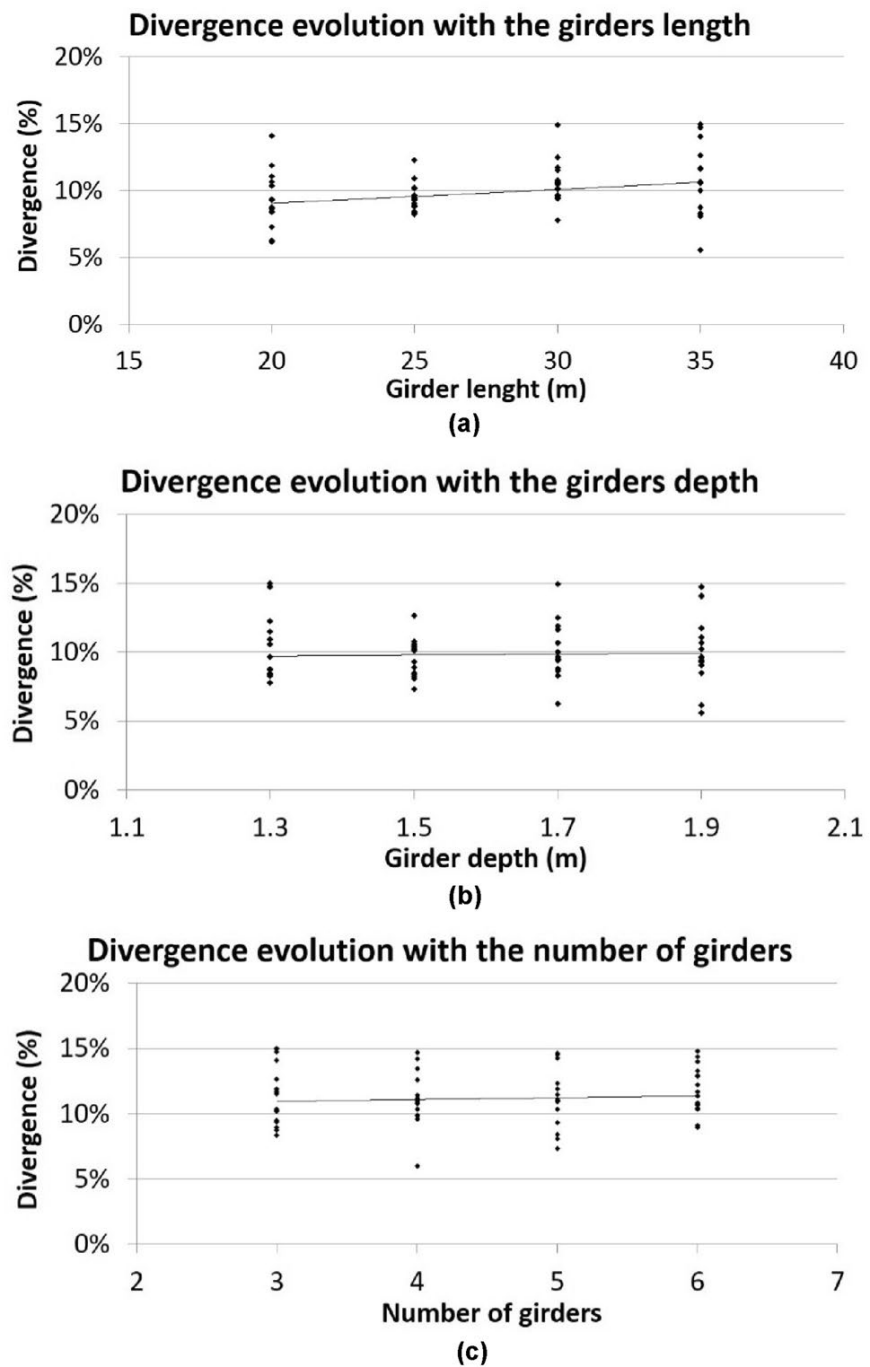
Divergence evolution with each of the analyzed variables: (**a**) girders length; (**b**) girders depth; (**c**) number of girders.

Figure 7 shows the evolution of the divergence between the calculation model proposed by the authors and the traditional structural grillage methods with the different variables analysed in the parametric study. The points shown in the graphs of Fig. 4 represent the divergence for each of the girder bridge deck models analysed in the parametric study. From the analysis of the evolution of the divergence with each of the variables analysed, the following can be concluded (a) the divergence increases with the length of the girder bridge deck; (b) the divergence increases with the depth of the girder bridge deck; (c) the divergence increases with the number of girders composing the bridge deck.

## Validation of the proposed method in real operational case

**Figure 8:**
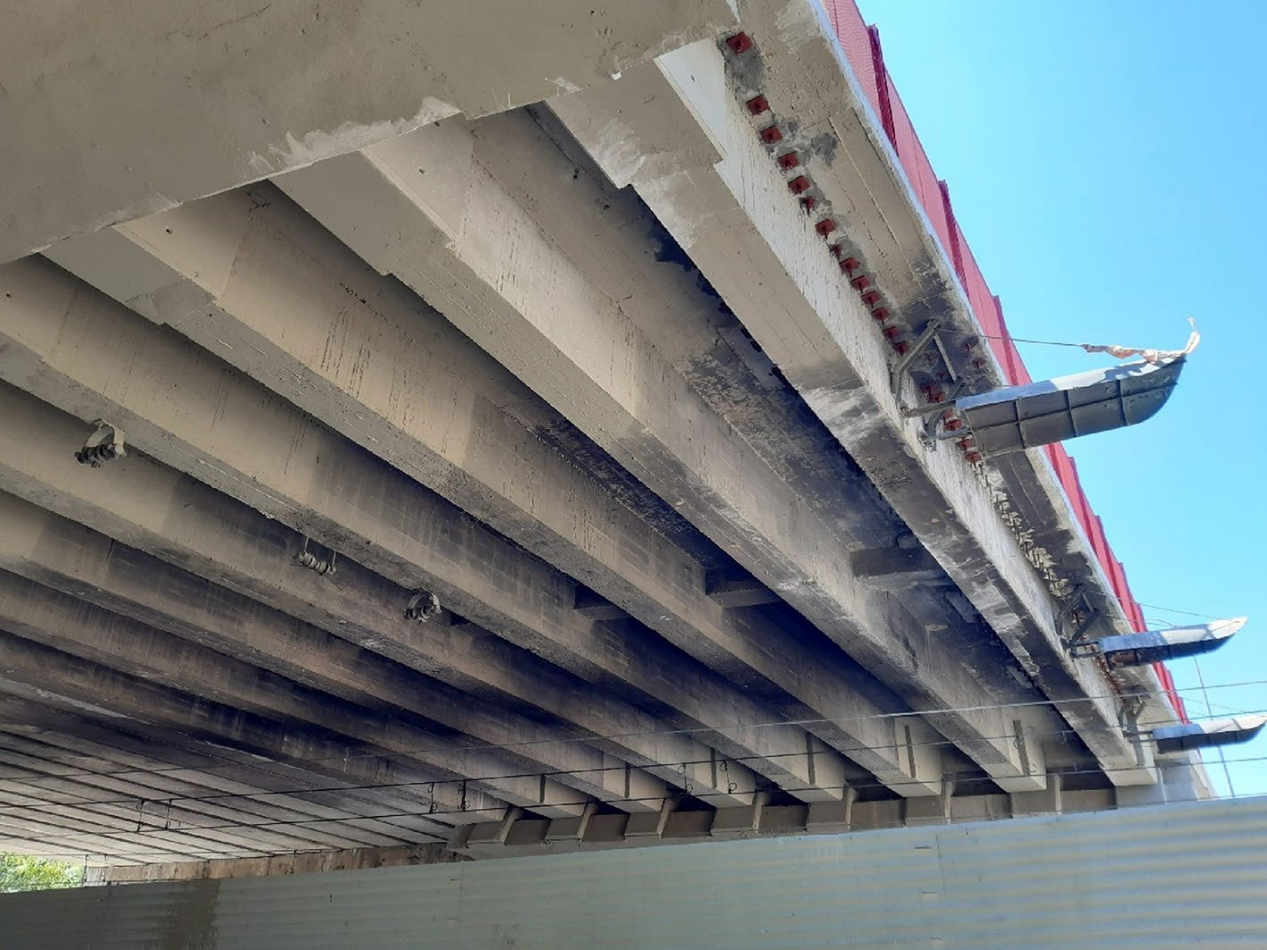
Road bridge analyzed.

**Figure 9:**
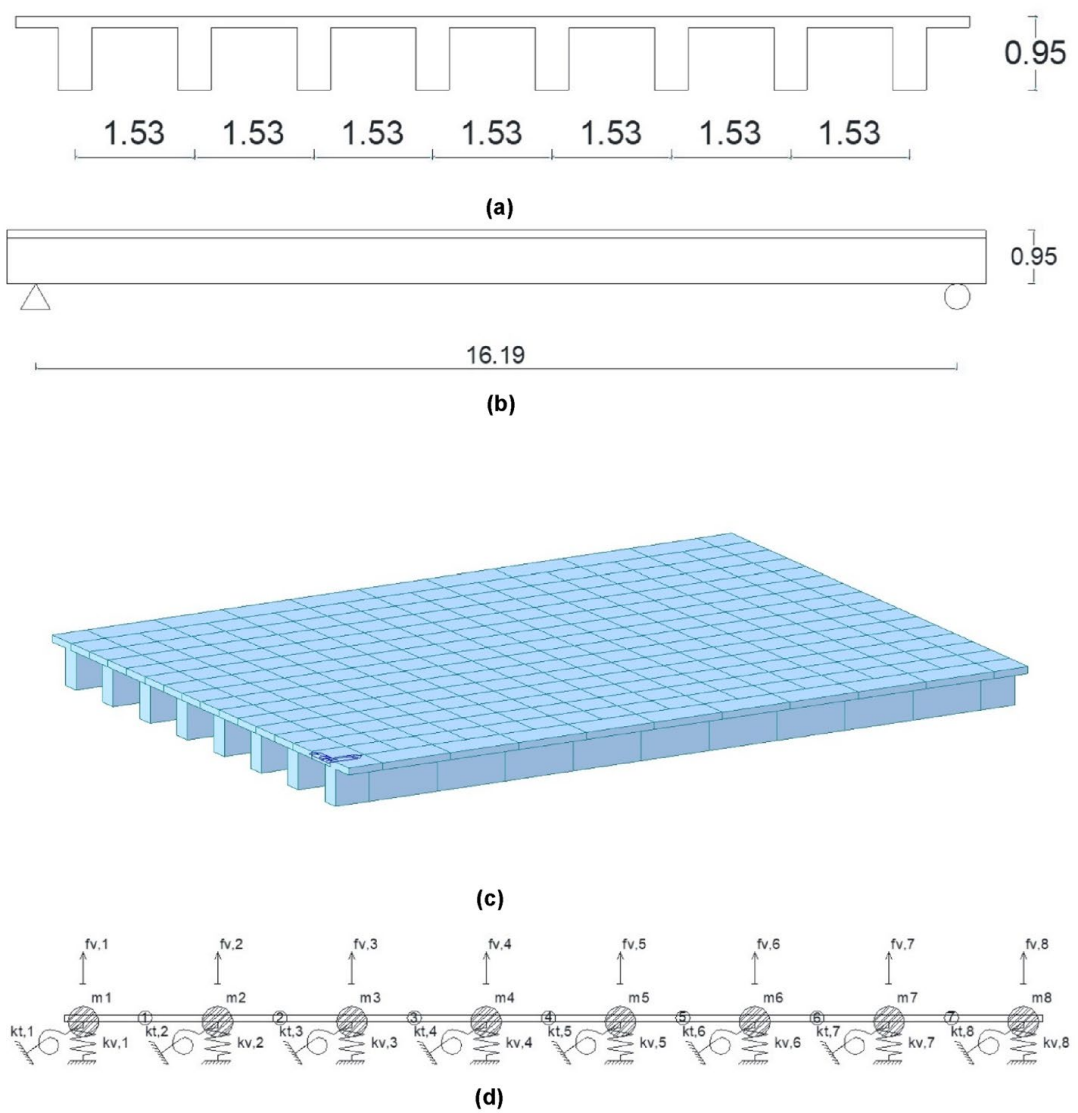
Structural model of the girder deck analyzed: (**a**) cross-section; (**b**) longitudinal profile; (**c**) traditional grillage model; (**d**) structural model proposed.

**Figure 10:**
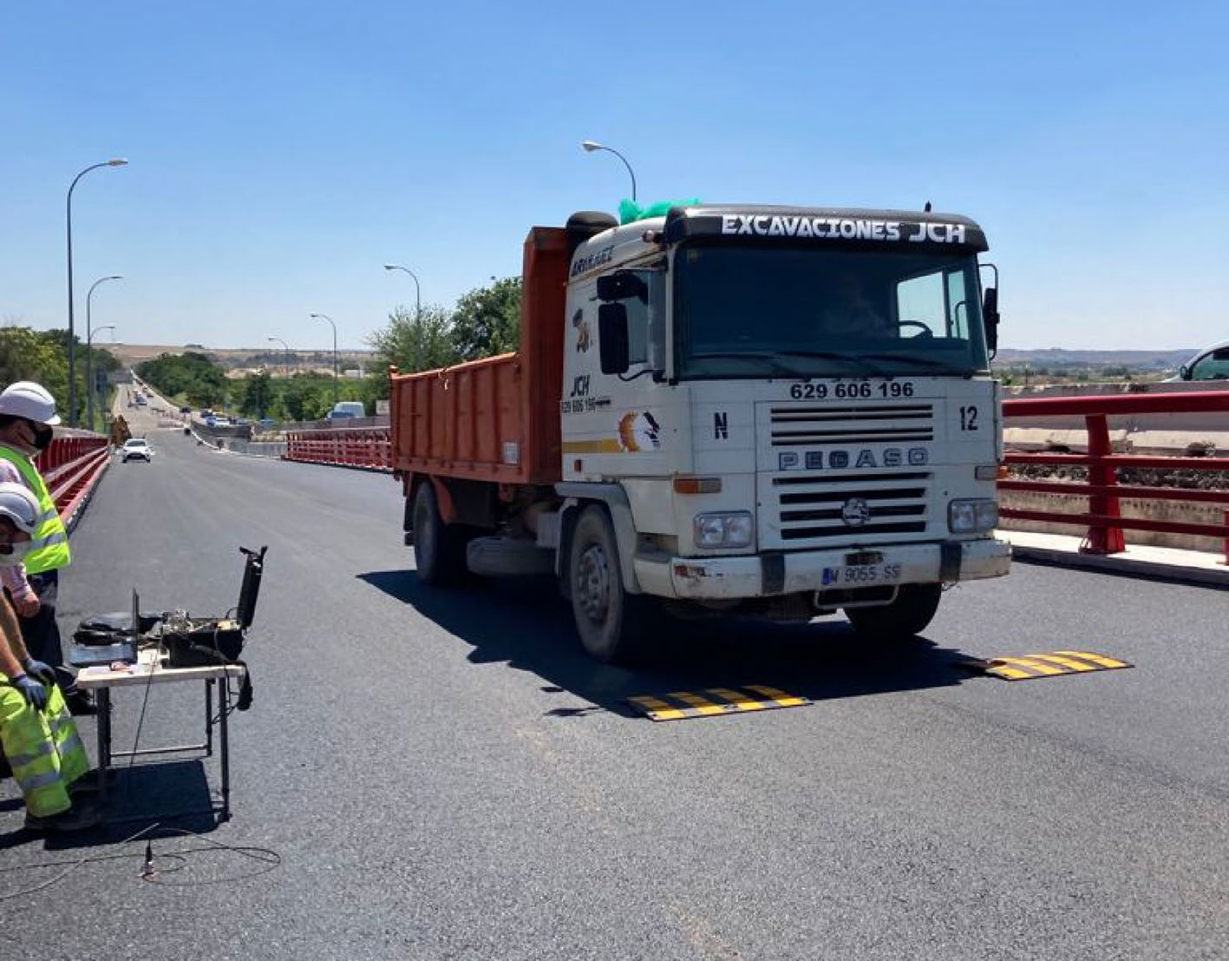
Truck passing over Rilem plate.

In this study, the dynamic load test of a road bridge (Fig. 8) is proposed for the validation of the method proposed by the authors for the modal analysis of girder bridge decks. The dynamic load test was carried out on a girder bridge located in Madrid, Spain. The girder bridge deck was built in situ, with a longitudinal support span of 16.19 m, and a cross section consisting of 8 beams of 0.80 m depth and 43 cm width, and a top slab of 15 cm thickness. The distance between the axes of the girders is 153 cm (Fig. 9). The excitation of the structure during the dynamic load test is performed by the passage of a two-axle truck with a total weight of 200 kN, and a wheelbase of 5.01 m. The truck passes over the structure four times during the dynamic load test. Four passes of the truck over the bridge deck are performed: (1) passage of the truck at a speed of 5–10 km/h; (2) passage of the truck at a speed of 30–40 km/h; (3) passage of the truck at a speed of 60 km/h; (4) passage of the truck at a speed of 10 km/h, passing the truck over a Rilem plate located in the central span of the girder bridge deck^25^ (Fig. 10).

### Instrumentation and monitoring arranged

**Figure 11:**
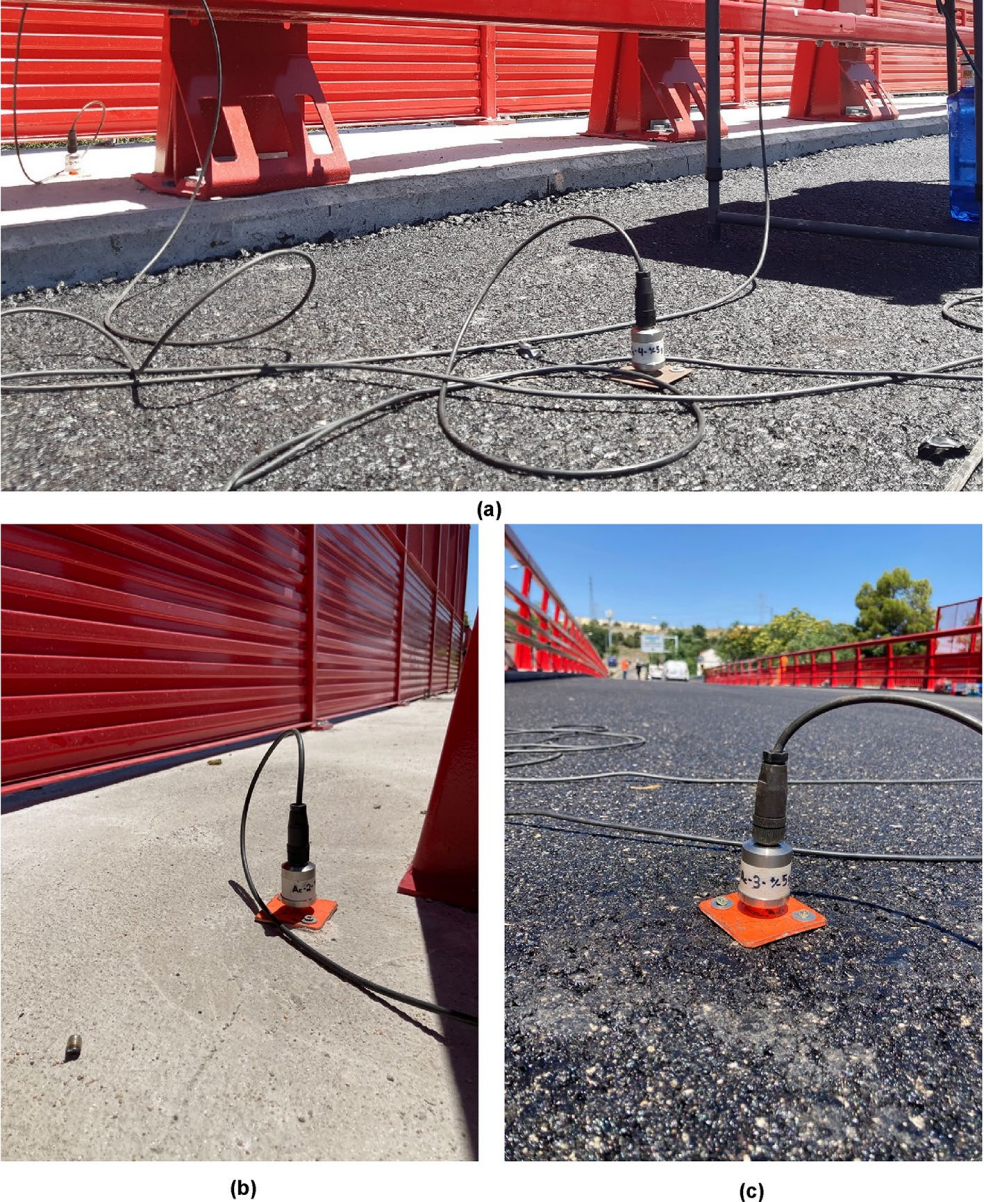
Piezoelectric accelerometers used in road bridge dynamic load test: (**a**) piezoelectric accelerometers on girders 1 and 2; (**b**) piezoelectric accelerometer on girder 8; (**c**) piezoelectric accelerometer on girder 7.

**Figure 12:**
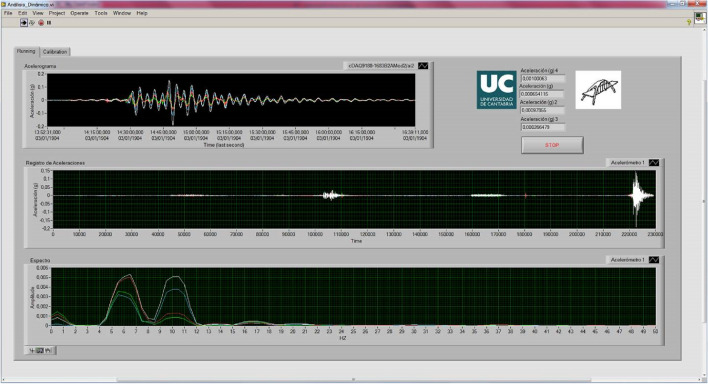
Real-time visualization of the data provided by the accelerometers.

To empirically characterise the structural response of the girder bridge deck during the dynamic load test, the authors installed a total of four piezoelectric accelerometers model "Metra KS48C" that provide the acceleration experienced by the two end girders on each side of the bridge deck width (Fig. 11). The four accelerometers are installed in the same cross-section of the deck, located at a distance of 1 m from the centre of the span of the girder bridge deck. The acquisition, recording and monitoring of the data provided by the sensors is performed by a Structural Health Monitoring System (SHMS) consisting of the following elements: (a) a Modular Central Data Acquisition and Processing Unit (MCDA&PU) model "NI-CDAQ-9188" with capacity to simultaneously manage the signal from up to eight Data Acquisition Units (DAU); (b) an acceleration DAU model "NI-9234" that facilitates the processing of the analogue signal coming from the accelerometers; (c) a workstation in charge of communicating with the MCDA&PU and of recording and visualising the data provided by the sensors through a Data Acquisition and Monitoring Program designed and programmed by the authors (Fig. 12).

### Results obtained

**Figure 13:**
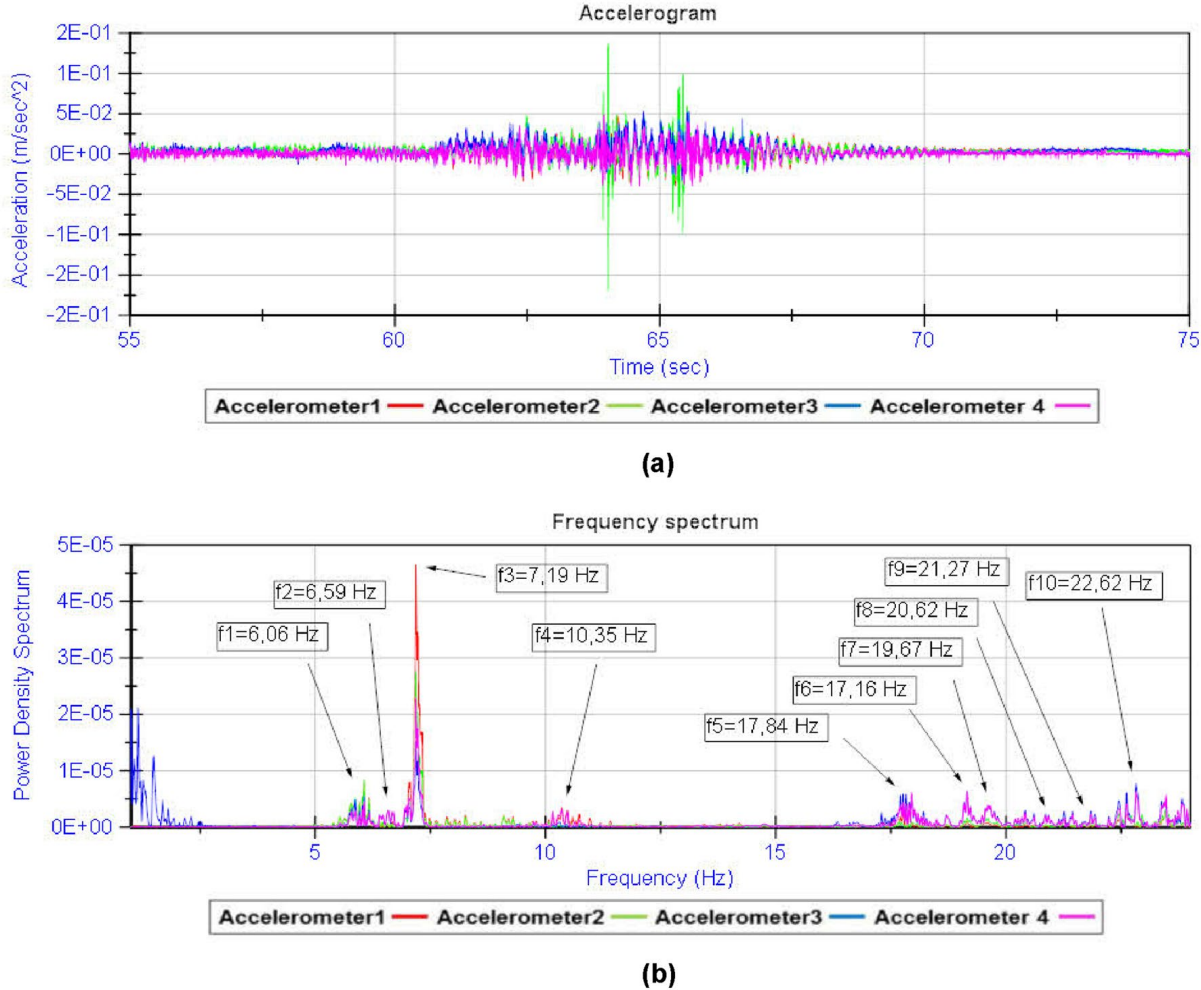
Analysis of the accelerations experienced by the girder bridge deck during the dynamic load test: (**a**) acceleration record; (**b**) frequency spectrum.

**Figure 14:**
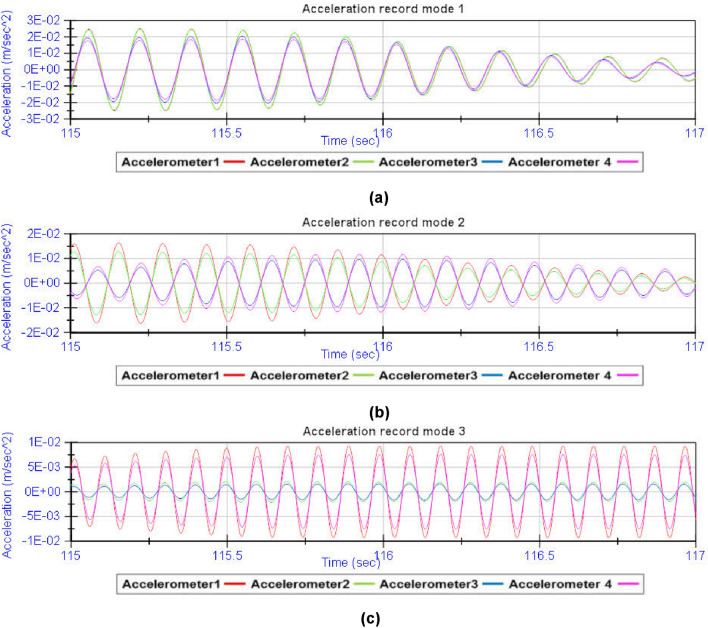
Spectral decomposition: (**a**) acceleration associated with vibration mode 1; (**b**) acceleration associated with vibration mode 2; (**c**) acceleration associated with vibration mode 3.

**Figure 15:**
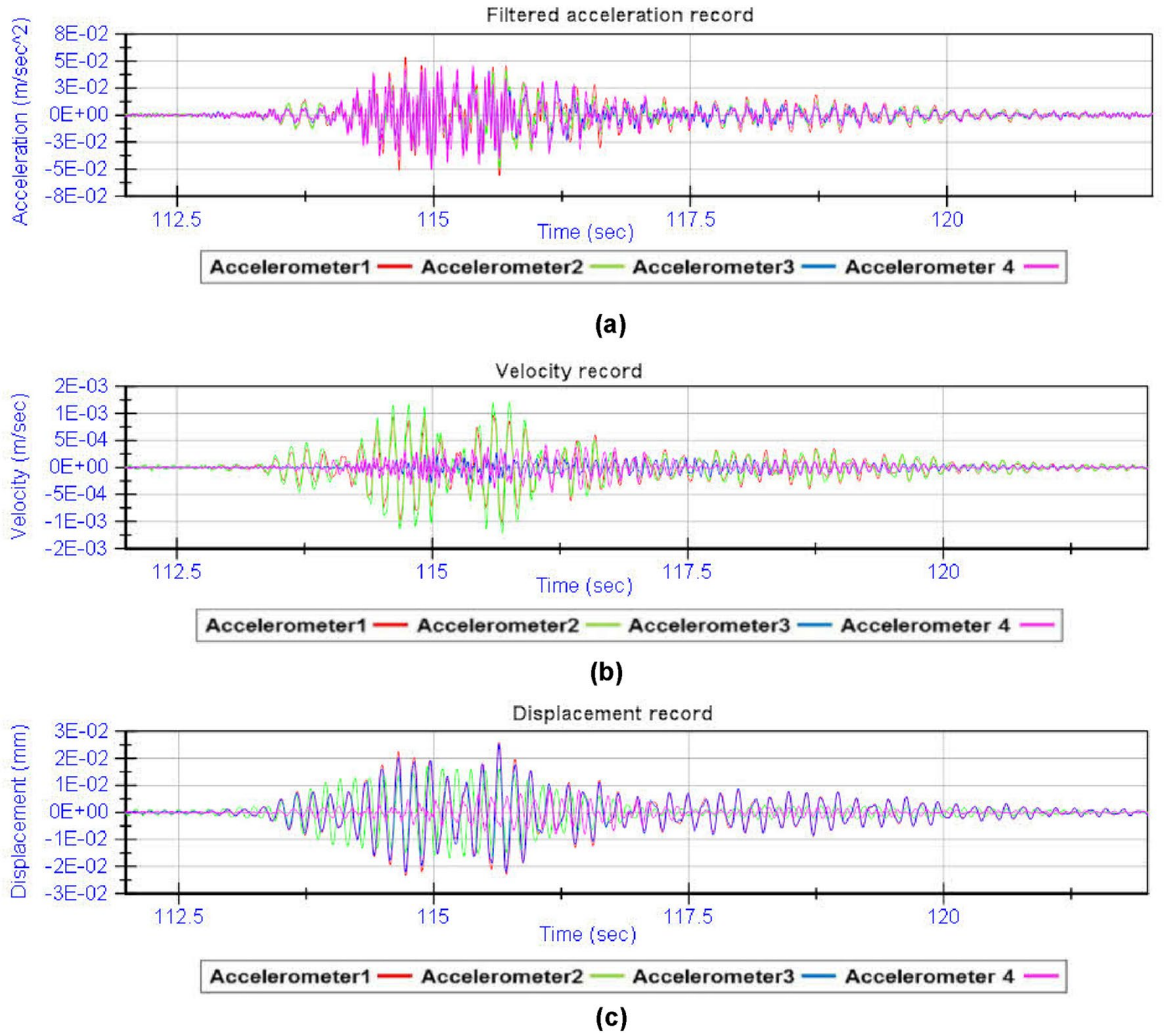
Acceleration filtering and numerical integration: (**a**) filtered acceleration record; (**b**) velocity record; (**c**) dynamic displacement record.

**Figure 16:**
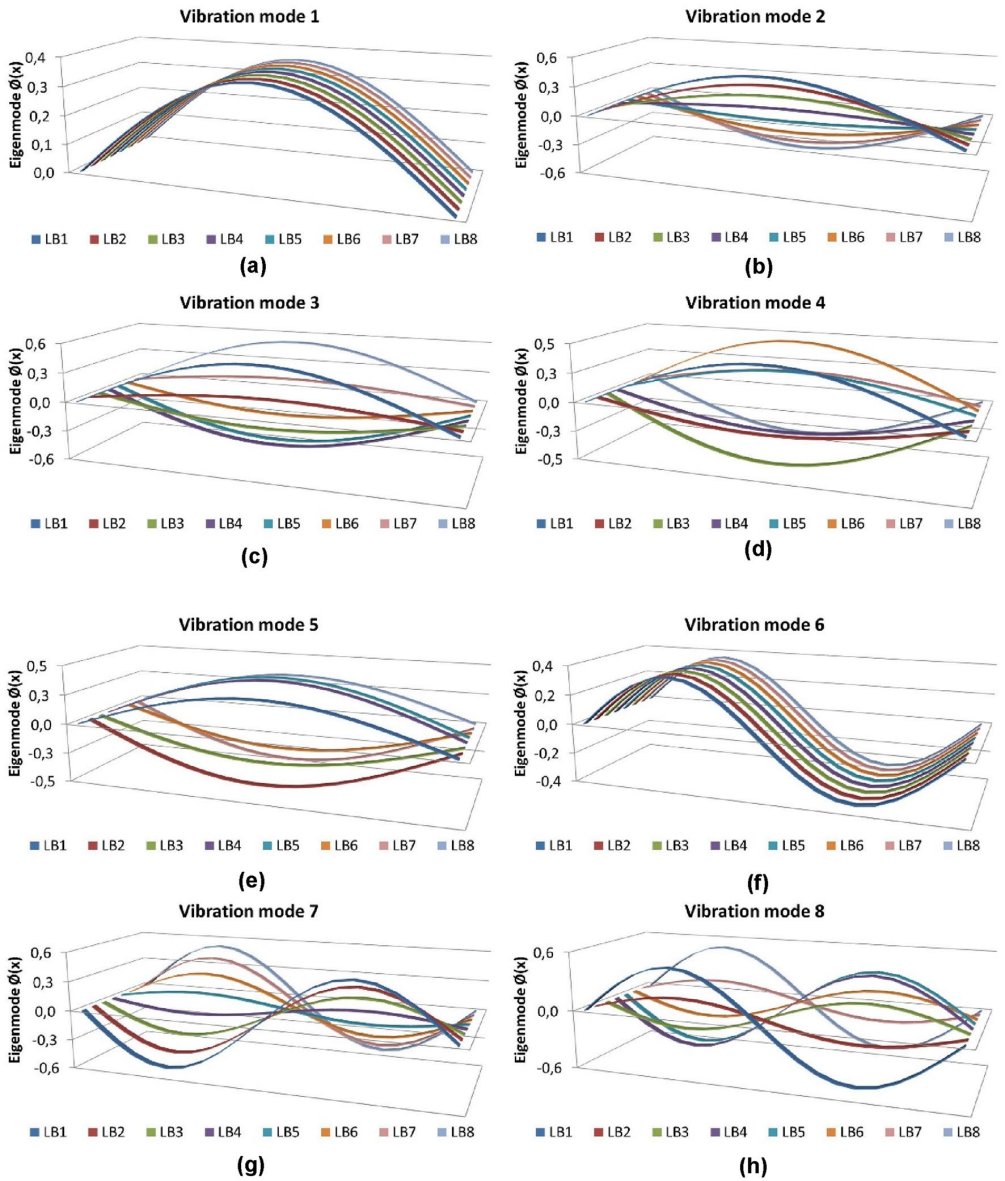
Modal analysis using the methodology proposed by the authors: (**a**) eigenmode function 1; (**b**) eigenmode function 2; (**c**) eigenmode function 3; (**d**) eigenmode function 4; (**e**) eigenmode function 5; (**f**) eigenmode function 6; (**g**) eigenmode function 7; (**h**) eigenmode function 8.

**Figure 17:**
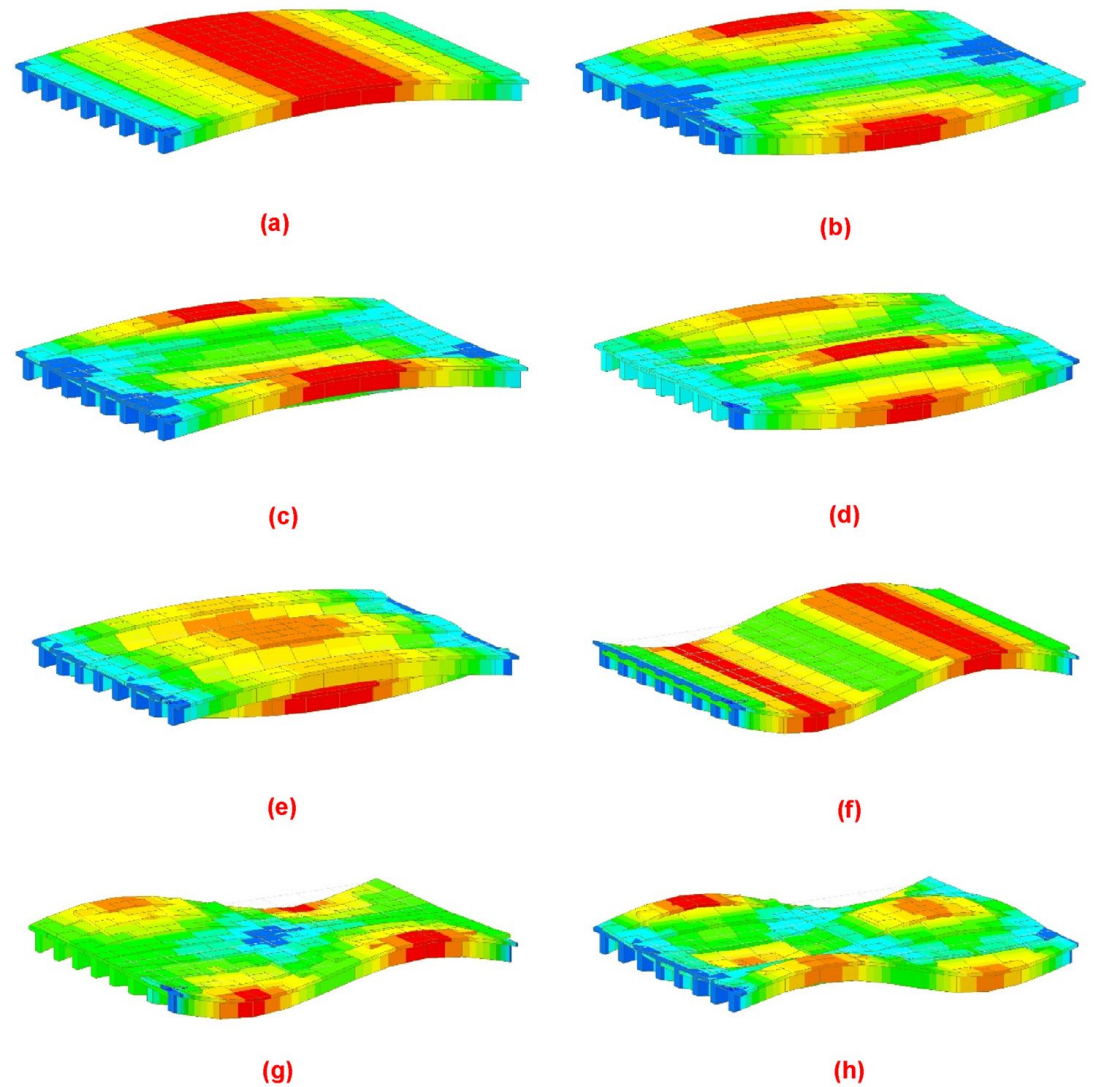
Modal analysis using a traditional grillage calculation model: (**a**) eigenmode function 1; (**b**) eigenmode function 2; (**c**) eigenmode function 3; (**d**) eigenmode function 4; (**e**) eigenmode function 5; (**f**) eigenmode function 6; (**g**) eigenmode function 7; (**h**) eigenmode function 8.

By analysing the acceleration record provided by the four accelerometers during the dynamic load test, the fundamental frequencies of vibration of the structure are obtained (Fig. 13). From the spectral decomposition and filtering of the raw acceleration record provided by the accelerometers, the shape functions associated with the fundamental modes of vibration of the girder bridge^26–29^ can be obtained (Figs. 14 and 15). The results obtained from the experimental analysis of the dynamic load test are compared with the theoretical modal analysis using the methodology proposed by the authors (Fig. 16) and the traditional grillage calculation methods (Fig. 17). The eigenmodes of the structure with a high vibration frequency are damped very quickly, in a small time interval, so the empirical characterisation of these vibration modes during the dynamic load test is more complicated and less accurate. The divergence between the natural vibration frequencies of the structure below 10 Hz obtained by the proposed methodology and those obtained experimentally is less than 10% (Table 3).

**Table 3:** Theoretical-empirical comparative analysis.

	Experimental dynamic load test	Proposed method		Grillage calculation model	
		Frequency	Divergence (%)	Frequency	Divergence (%)
f1 (Hz)	6.060	5.704	6	5.675	6
f2 (Hz)	6.590	5.891	11	6.199	6
f3 (Hz)	7.190	7.763	8	8.362	16
f4 (Hz)	10.350	12.413	20	11.544	12
f5 (Hz)	17.840	20.545	15	17.083	4
f6 (Hz)	19.160	22.270	16	21.327	11
f7 (Hz)	19.670	22.238	13	21.535	9
f8 (Hz)	20.420	24.949	22	24.306	19

## Summary and conclusions

The proposed method has been presented, parametrically discussed and validated on a real operational bridge, and the divergence of the method from traditional computational methods has been analysed. The parametric study considers the length, number and depth of the girders. The modal analysis of a total of 64 girder bridge decks is presented. The variables analysed are the natural frequencies and eigenmodes of the girder bridge deck. The results of the parametric study show an average divergence between the proposed method and the traditional ones of 10%, the average divergence in the fundamental mode of vibration being less than 3%. A divergence value of 3% in the fundamental frequency of vibration corresponds to a difference between the stiffnesses of the structural models of less than 6%. Considering that the overestimation of the load associated with the extreme beams assumed in simplified methods using Load Distribution Factors (LDF) ranges from 125 to 300%^30–33^, it is considered that the method proposed by the authors adequately reproduces the modal structural response of girder bridge decks. Furthermore, the results of the proposed method are experimentally contrasted by a dynamic load test of a full-scale girder bridge where the divergence between the experimental dynamic structural response and the one obtained by applying the proposed method during the dynamic load test of the girder bridge is analysed. The divergence between the natural frequencies of vibration of the structure below 10 Hz obtained experimentally and that obtained theoretically by applying the method proposed by the authors is less than 10%.

Currently, different types of data sources (sensory and synthetic) coexist, but their full potential is not exploited due to poor connectivity. Thus, on the one hand, we make the comparison of different data a reality and accelerate the generation of efficient models. All this information could feed a bridging DT model to keep the latest information up to date. This method could help to implement a Decision Support System (DSS) and improve usability and accuracy in mission configuration and safe manual navigation from the road control centre by integrating vehicle-to-infrastructure communication (V2I, i.e. truck platooning applications and traffic management) to capture data and process it through artificial intelligence AI analysis. The simplicity of the method allows it to be easily integrated into optimal bridge design strategies^34^ and more heuristic approaches^35–40^ to cope intelligently with today's competitive world.

Nowadays, the increasing traffic demands on communication networks together with the exposure to variations in environmental variables, make the condition assessment of structures a necessity to avoid an uncontrolled increase in system failures and unexpected downtime, while keeping the costs associated with maintenance and inspection at reasonably low levels^41^. During the last few years, research on DT technology applied to civil engineering or building structures has increased significantly, especially in the areas of operation and maintenance (O&M). For decades, structural health monitoring (SHM) systems have produced large amounts of monitoring data to control structural behaviour during operation^42^. With the expansion of AI applications to engineering problems, this data can be processed by algorithms capable of extracting information relevant to the current and future health status of structures^43^ sometimes hiding the physical correlation^44,45^. The latter, together with the maturity of physics-based structural response models achieved over decades, have opened the door to new condition assessment paradigms that merge data and model information under a more digital-centric trend.

## Data Availability

The data that support the findings of this study are available from the corresponding author, Gaute A., upon reasonable request.
